# Exome sequencing reveals neurodevelopmental genes in simplex consanguineous Iranian families with syndromic autism

**DOI:** 10.1186/s12920-024-01969-6

**Published:** 2024-08-05

**Authors:** Mohammad-Reza Ghasemi, Hossein Sadeghi, Farzad Hashemi-Gorji, Reza Mirfakhraie, Vijay Gupta, Afif Ben-Mahmoud, Saman Bagheri, Katayoon Razjouyan, Shadab Salehpour, Seyed Hassan Tonekaboni, Mehdi Dianatpour, Davood Omrani, Mi-Hyeon Jang, Lawrence C. Layman, Mohammad Miryounesi, Hyung-Goo Kim

**Affiliations:** 1 Department of Medical Genetics, Faculty of Medicine, Shahid Beheshti University of Medical Sciences, Tehran, Iran; 2 Center for Comprehensive Genetic Services, Shahid Beheshti University of Medical Sciences, Tehran, Iran; 3 Genomic Research Center, Shahid Beheshti University of Medical Sciences, Tehran, Iran; 4 Neurological Disorders Research Center, Qatar Biomedical Research Institute, Hamad Bin Khalifa University, Doha, Qatar; 5 Psychiatric Department, Shahid Beheshti University of Medical Sciences, Tehran, Iran; 6 Department of Pediatric Endocrinology & Metabolism, School of Medicine, Loghman Hakim Hospital, Shahid Beheshti University of Medical Sciences, Tehran, Iran; 7 Department of Pediatric Neurology, School of Medicine, Pediatric Neurology Research Center, Mofid Children’s Hospital, Shahid Beheshti University of Medical Sciences, Tehran, Iran; 8 Department of Medical Genetics, Faculty of Medicine, Shiraz University of Medical Sciences, Shiraz, Iran; 9 Stem Cells Technology Research Center, Shiraz University of Medical Sciences, Shiraz, Iran; 10 Department of Neurosurgery, Robert Wood Johnson Medical School, The State University of New Jersey, Rutgers, Piscataway, NJ 08854 USA; 11 Section of Reproductive Endocrinology, Infertility and Genetics, Department of Obstetrics and Gynecology, Augusta University, Augusta, GA 30912 USA; 12 Department of Neuroscience and Regenerative Medicine, Medical College of Georgia, Augusta University, Augusta, GA 30912 USA

**Keywords:** Syndromic autism, Exome sequencing, Neurodevelopmental disorder, Muscular dystrophy, *De novo* variants, Consanguineous simplex family, Iran

## Abstract

**Background and objective:**

Autosomal recessive genetic disorders pose significant health challenges in regions where consanguineous marriages are prevalent. The utilization of exome sequencing as a frequently employed methodology has enabled a clear delineation of diagnostic efficacy and mode of inheritance within multiplex consanguineous families. However, these aspects remain less elucidated within simplex families.

**Methods:**

In this study involving 12 unrelated simplex Iranian families presenting syndromic autism, we conducted singleton exome sequencing. The identified genetic variants were validated using Sanger sequencing, and for the missense variants in *FOXG1* and *DMD*, 3D protein structure modeling was carried out to substantiate their pathogenicity. To examine the expression patterns of the candidate genes in the fetal brain, adult brain, and muscle, RT-qPCR was employed.

**Results:**

In four families, we detected an autosomal dominant gene (*FOXG1*), an autosomal recessive gene (*CHKB*), and two X-linked autism genes (*IQSEC2* and *DMD*), indicating diverse inheritance patterns. In the remaining eight families, we were unable to identify any disease-associated genes. As a result, our variant detection rate stood at 33.3% (4/12), surpassing rates reported in similar studies of smaller cohorts. Among the four newly identified coding variants, three are *de novo* (heterozygous variant p.Trp546Ter in *IQSEC2*, heterozygous variant p.Ala188Glu in *FOXG1*, and hemizygous variant p.Leu211Met in *DMD*), while the homozygous variant p.Glu128Ter in *CHKB* was inherited from both healthy heterozygous parents. 3D protein structure modeling was carried out for the missense variants in *FOXG1* and *DMD*, which predicted steric hindrance and spatial inhibition, respectively, supporting the pathogenicity of these human mutants. Additionally, the nonsense variant in *CHKB* is anticipated to influence its dimerization – crucial for choline kinase function – and the nonsense variant in *IQSEC2* is predicted to eliminate three functional domains. Consequently, these distinct variants found in four unrelated individuals with autism are likely indicative of loss-of-function mutations.

**Conclusions:**

In our two syndromic autism families, we discovered variants in two muscular dystrophy genes, *DMD* and *CHKB*. Given that *DMD* and *CHKB* are recognized for their participation in the non-cognitive manifestations of muscular dystrophy, it indicates that some genes transcend the boundary of apparently unrelated clinical categories, thereby establishing a novel connection between ASD and muscular dystrophy. Our findings also shed light on the complex inheritance patterns observed in Iranian consanguineous simplex families and emphasize the connection between autism spectrum disorder and muscular dystrophy. This underscores a likely genetic convergence between neurodevelopmental and neuromuscular disorders.

## Introduction

Autism spectrum disorder (ASD) is a group of neurodevelopmental conditions characterized by the triad of communication deficit, abnormal social interest, and restricted/repetitive behavior [1, 2]. While non-syndromic autism pertains to cases where autism stands as the primary diagnosis, syndromic autism manifests additional clinical features such as intellectual disability, epilepsy, craniofacial anomalies or muscular dystrophy, usually associated with chromosomal abnormalities or single-gene mutations [3].

The most comprehensive genetic progress has been made in gene discovery of syndromic autism, encompassing more than 1,000 genes or gene loci thus far [4]. This substantial genetic diversity poses direct challenges in the identification of causative variants and the interpretation of genetic test results within clinical settings [3]. Though these genes represent a minority within the spectrum of autism cases, studies have demonstrated that syndromic and non-syndromic forms share common biological pathways such as synaptic maintenance and transcriptional regulation [4], chromatin remodelling [5], and ubiquitination [6]. Gene discovery in individuals with autism will provide tremendous insight into underlying pathogenic mechanisms. Recent discoveries have also underscored the significance of *de novo* mutations (DNMs) underlying syndromic autism. As such, the risk of autism emerges from numerous rare *de novo* and inherited variants, rather than a limited number of common variants [7].

Confronted with the intricate genetic locus heterogeneity and clinical diversity inherent in this diverse group of disorders, single gene testing in autism faces one of its most formidable challenges. The advent of exome sequencing (ES) in human genetics has emerged as a potent molecular tool for investigating individuals afflicted by the broad spectrum of hitherto undiagnosed syndromic forms [8].

Noteworthy, multiplex consanguineous families grappling with neurodevelopmental disorders have proven invaluable resources in human molecular genetics, playing a critical role in uncovering novel autosomal recessive disease genes through exome sequencing (ES) [9–11]. In contrast, the diagnostic yield and inheritance patterns in simplex consanguineous families, particularly within the Iranian context, remain ambiguously defined.

This present study delves into the genetic underpinning of syndromic autism through a phenotype-first approach, set against the backdrop of a well-defined clinical syndrome [3]. Syndromic autism opens fresh avenues for comprehending both molecular and phenotypic heterogeneity. We aimed to unravel the inheritance pattern and interrelationship of coexisting clinical features across 12 simplex consanguineous Iranian families, by identifying disease genes associated with a range of phenotypes, including muscular dystrophy.

## Materials and methods

### Human subjects

**Fig. 1:**
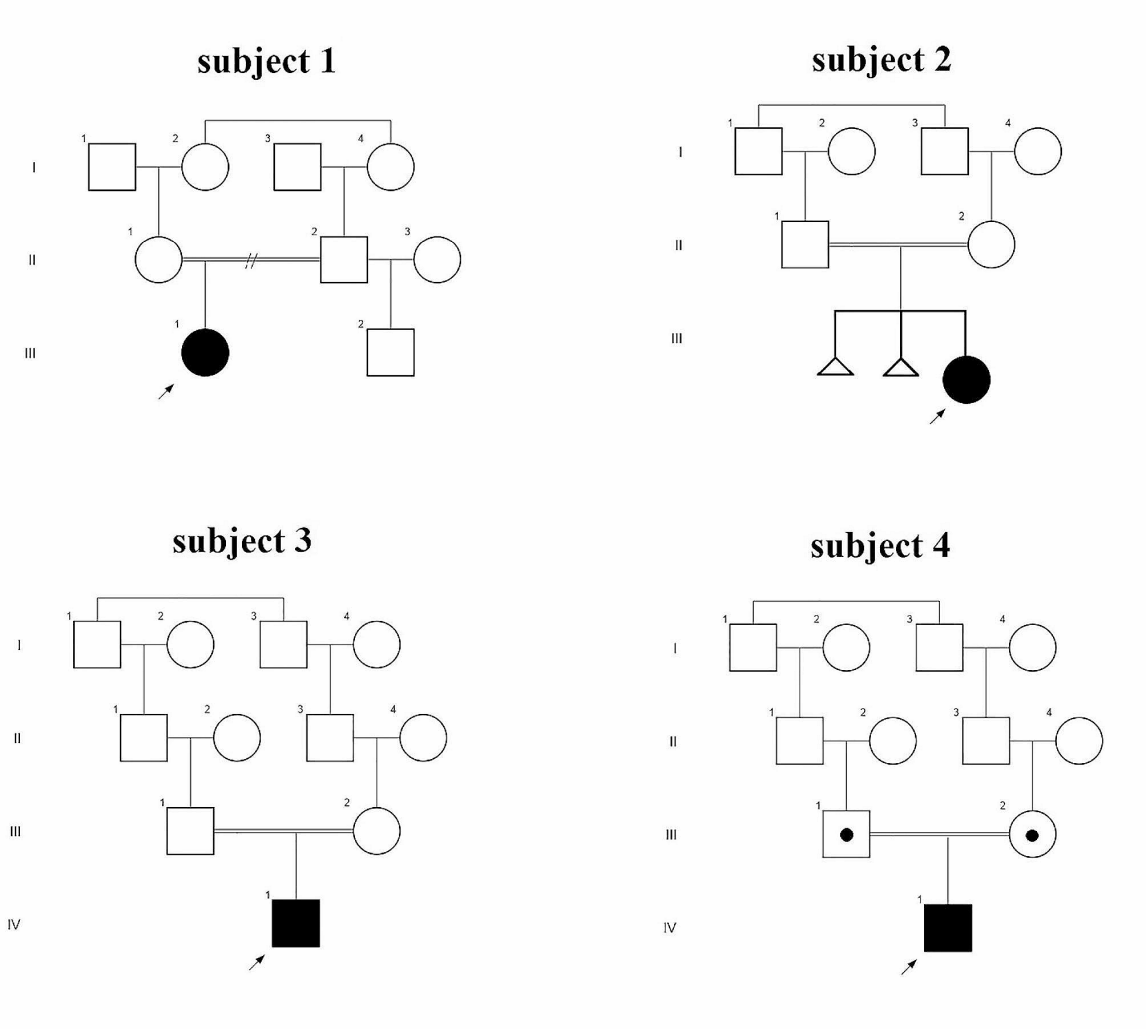
Pedigrees of four simplex families. Pedigrees of four families showing affected females (solid circles) and males (solid squares). Carrier parents are indicated by open circles/squares with black dots

The study sample consisted of twelve Iranian families diagnosed with ASD. They were referred to the outpatient clinical genetic department by psychiatric specialists for ES. Prior to ES, karyotype analysis and microarray tests were performed on four individuals from these families to identify chromosome anomalies and CNVs. The results were negative [12]. A team of specialists from the departments of neurology, pediatrics, and psychiatry, collaboratively working in a joint clinic, conducted comprehensive evaluations on all four individuals, ultimately confirming the diagnosis of syndromic autism. Following written informed consent from the families, complete clinical assessments and pedigree information were gathered prior to ES (Fig. 1).

### DNA extraction

To identify the genetic defects in the affected members, genomic DNA (gDNA) was extracted from peripheral blood samples of probands and their parents using the salting out method. The concentration and quality of gDNA were assessed with a NanoDrop 1000 (Thermo Fisher Scientific, Inc., Wilmington, DE, USA).

### Exome sequencing

Custom-designed Nimblegen chips were utilized for capturing and next-generation sequencing to perform ES. Briefly, after shearing one µg of gDNA from the proband, the SureSelectXT2 V6 kit was used for the enrichment of the targeted sequence, followed by paired-end sequencing on an Illumina HiSeq4000 with read length 101 bp and coverage 100X.

### Data analysis

Manufacturer’s proprietary software-generated read files (Fastq) were generated from the sequencing platform. To remove the adaptor and low-quality reads, the Fastq file was trimmed and aligned to human reference genome hg19 by recruiting Burrows-Wheeler Aligner (BWA). BAM files were then evaluated for variant calling by using SAMtools and Picard, followed by variant annotation via the ANNOVAR software [13–17].

We utilized a comprehensive set of techniques to evaluate the pathogenicity of variants identified in the VCF files. Initial filtering was performed by determining variant frequency in public databases, including the Mouse Genome Database (MGD), OMIM, PubMed, and ClinVar. Our final selection of variants was guided by several key factors. These included adherence to ACMG guidelines, non-occurrence in control datasets like gnomAD (https://gnomad.broadinstitute.org/), dbSNP138 (https://www.ncbi.nlm.nih.gov/snp/), 1000 Genome projects (http://www.internationalgenome.org), and the Iranome database [18], significance of interacting proteins in neurodevelopmental disorders and intellectual disability as reported in scientific literature, occurrence of sporadic variants in the Human Gene Mutation Database (HGMD), expression profiles in various human tissues, and the phenotypes noted in existing knock-out/deletion model organisms. To generate the initial list of candidate variants, we applied a series of filtering steps. These steps involved including only variants with a minor allele frequency (MAF) less than 0.01 in ExAC/gnomAD v2.11/1000g2015, focusing on exonic, splicing, non-synonymous, and stopgain variants with sufficient coverage. Variants, whether rare and novel, were prioritized according to nonsynonymous, indel, and putative splice sites. To predict the pathogenicity of the candidate variant, predictor tools including Polyphen2 (http://genetics.bwh.harvard.edu/pph2/), SIFT (https://sift.bii.a-star.edu.sg/), MutationTaster (http://www.mutationtaster.org/), and most importantly CADD software (≥ 20), were employed for *in silico* evaluation [19–23]. To assess the impact of missense variants on the structure and functionality of our candidate genes, we employed protein modeling. The ultimate sequencing results were also analyzed alongside international mutation and polymorphism databases, in addition to the in-house database.

### Wechsler Intelligence Scale for Children (WISC)

The Wechsler Intelligence Scale for Children (WISC) was utilized to evaluate the intellectual abilities of participants. The WISC, a well-established psychological assessment tool developed by David Wechsler, is designed to measure cognitive abilities across various domains. It includes core subtests that assess areas such as Verbal Comprehension, Perceptual Reasoning, Working Memory, and Processing Speed. These subtests are pivotal in determining the Full-Scale IQ (FSIQ), which reflects the overall cognitive ability of a child. The assessment also allows for the use of supplemental subtests to gain additional insights into a child’s cognitive profile.

Central to our analysis was the WISC composite score range, which is standardized with a mean score of 100 and a standard deviation of 15. The scores span from 40 to 160, categorizing intellectual capabilities into different classifications such as ‘Very Superior’ (130 and above), ‘Superior’ (120–129), ‘High Average’ (110–119), ‘Average’ (90–109), ‘Low Average’ (80–89), ‘Borderline’ (70–79), and ‘Extremely Low’ (below 70) [24, 25].

### Sanger confirmation and segregation analysis

**Fig. 2:**
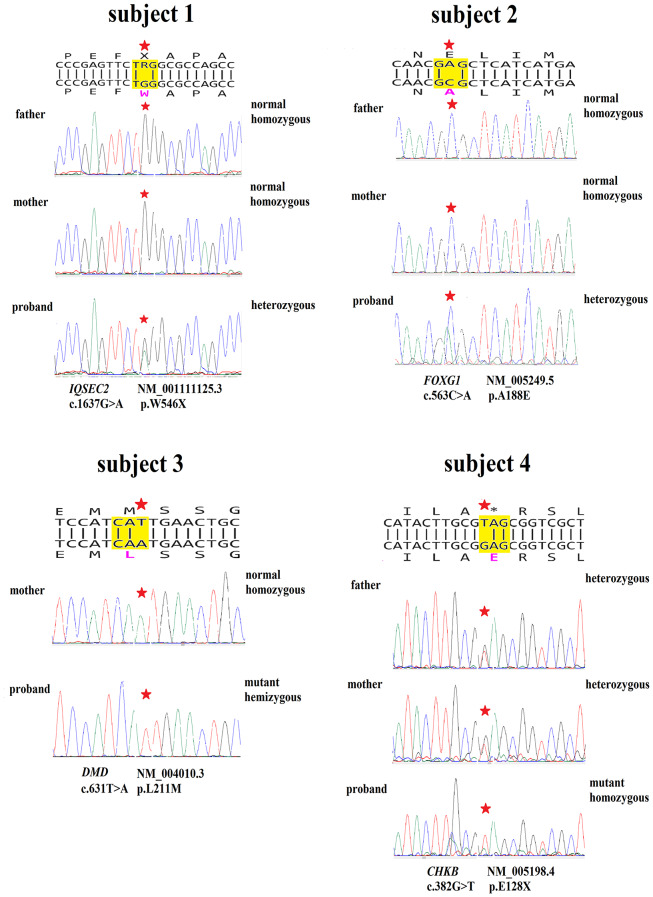
Genetic variants in four genes in Iranian families. The chromatograms display variants within the four genes across four simplex Iranian families, with variant positions indicated by asterisks

To confirm the ES results, flanking PCR primers (Table 1) were designed for all variants. Subsequent PCR products were then subjected to Sanger sequencing. Segregation analysis, using proband and parents’ samples, exhibited that only two heterozygous (*IQSEC2* and *FOXG1*) variants and one hemizygous variant in *DMD* in 3 families were *de novo*. Additionally, a homozygous variant in *CHKB* segregated in the family in an autosomal recessive fashion. The corresponding chromatograms are presented in Fig. 2.

**Table 1:** Sequences of primers used for segregation analysis

*IQSEC2*	Forward	5’- GCTTGAGTCTAAATGGACACAAAGG-3’	Intronic
	Reverse	5’- GGCACTGGTGGAGGAACT-3’	Exonic
*FOXG1*	Forward	5’- GGCCGGACGAGAAGGAGAAG-3’	Exonic
	Reverse	5’- GCGCGGTCCATGAAGGTGAG-3’	Exonic
*DMD*	Forward	5’- TCCCTATTGTCTGTATCTGCTG-3’	Intronic
	Reverse	5’- AATGCCTTCAATGGTTGCTCTC-3’	Intronic
*CHKB*	Forward	5’- ACCTCATTACACCGAAGCCT-3’	Intronic
	Reverse	5’- GCACCCAGGAAGCTATCAG-3’	Intronic

### RT-qPCR analysis

Total RNA extracted from human brain and other tissues (Human Total RNA Master Panel II, Cat# 636643, Clontech) was used for RT-qPCR. The catalogue number for eight brain tissues from Clontech are as follows: dorsal root ganglion-636,150, fetal brain total-636,526, substantia nigra total-636,560, cerebral cortex-636,561, occipital lobe- 636,570, parietal lobe-636,571, postcentral gyrus-636,573, hippocampus-636,565. For cDNA synthesis, 1 µg of total RNA was utilized with the high-capacity cDNA Reverse Transcription Kit. The resulting cDNA was then analyzed via RT-PCR on the QuantStudio 6 Flex system using SYBR Green (ThermoFisher, Waltham, MA). The primer sequence used for this analysis is presented in Table 2. Utilizing the comparative ΔCt method, the relative expression of each gene was calculated. In essence, the relative gene expression was determined by finding the difference (ΔCt) between the Ct value of the gene of interest and the reference gene, GAPDH. Subsequently, the fold change 2^(-ΔCt) was calculated, and the relative expression was visualized through Exel graphs.

**Table 2:** Sequences of primers used for qRT-PCR for four genes *IQSEC2*,* FOXG*,* DMD*, and *CHKB*

*IQSEC2*	Forward	5’- AGCGGGAGTCAAAGGAACAG − 3’
	Reverse	5’- GGGGAACTGTGTCATCCAGG − 3’
*FOXG1*	Forward	5’- GCCAAGTTTTACGACGGGAC − 3’
	Reverse	5’- AGGGTTGGAAGAAGACCCCT − 3’
*DMD*	Forward	5’- TGAGGGAACAGCTCAAAGGC − 3’
	Reverse	5’- CCTTCTGCAGTCTTCGGAGT − 3’
*CHKB*	Forward	5’- CCTGGTGCTAGAAAGCGTGA − 3’
	Reverse	5’- CCCTCTGGGAAGACTCCGTA − 3’

### Protein modeling

Protein modeling was conducted using Phyre2 [26] and SWISS-MODELING server [27]. The resulting 3D protein structure model was visualized using PyMol software (PyMOL Molecular Graphics System, Version 1.2r3pre, Schrödinger, LLC, USA), and for the analysis of point variant tolerance and structural effects in the proteins, the MetaDome webserver [28] was employed.

## Results

### Clinical description of human subjects

#### Subject 1

This individual is a 21-year-old Iranian female from a consanguineous marriage (first cousins), diagnosed with a Rett-like phenotype. She exhibited multiple comorbidities including intellectual disability, ASD, motor delay, and speech delay. Born full-term by spontaneous vaginal delivery, she had ordinary biometric parameters. Her perinatal and neonatal periods were uneventful. According to her parents, she did not walk, sit, or speak until 22 months. Her first word was spoken very late, and she hasn’t yet formed full sentences. However, she is currently able to express certain words. While her motor milestones were not normal, a frequent problem is bolting (running away), which has led to injuries. In her initial neuropsychological testing, her Wechsler Intelligence Scale for Children-Fifth Edition (WISC-V) full-scale score was extremely low, falling within a Composite Score Range of 69 and below. Poor performance was observed across visual-spatial, fluid reasoning, processing speed, working memory, and verbal comprehension indices. She continues to struggle with independent planning, organization, and frustration when faced with challenging tasks. There were concerns about neurobehavioral patterns, including repetitive movements and behavior. At the age of 2 years 2 months, she was diagnosed with ASD. Her social interactions are limited; she lacks friends, social skills, and tends to avoid crowds. Furthermore, a progressively significant anxiety disorder was detected. Five fundamental deficits were considered: acrophobia, attention deficit hyperactivity disorder (ADHD), astasia, asthenia, and fatigability (inability to sustain muscular force against resistance).

#### Subject 2

This proband is the only child of consanguineous healthy parents (first cousins). This individual is an 18-year-old Iranian female with a history of ASD, intellectual disability, obesity, and seizures. Born full-term by spontaneous vaginal delivery, she had average biometric parameters. From her first year of life, four fundamental deficits were identified: hypotonia, astasia/abasia, speech delay, and ADHD. Her first words were spoken in the second year of life, and full sentences did not emerge until the age of five. She currently speaks some ambiguous words. Her most recent neuropsychological testing demonstrated a very low WISC-V full-scale score falling within the Composite Score Range of 70–79. Poor performance was noted across working memory, visual-spatial, fluid reasoning, and processing speed indices, with low average performance in the verbal comprehension index. Neurobehavioral concerns were apparent from the very beginning, characterized by repetitive movements and behaviors. A diagnosis of atypical Rett syndrome with multiple comorbidities was established (Table 3). During her childhood, several events happened, including seizures without fever. Complex partial seizures were diagnosed which have been well controlled with sodium valproate. Significant obesity is evident with a BMI exceeding 30 for the past five years. This trend continues due to an unhealthy lifestyle.

#### Subject 3

This individual is a 16-year-old boy with a history of muscular dystrophy and autism. He was born full-term by spontaneous vaginal delivery with normal biometric parameters from consanguineous healthy parents (second cousins). Although he had hypotonia in his first year of life, particularly concerning his head and neck control, his motor milestones during childhood were initially typical, yet they have gradually declined over time. He has been diagnosed with Duchenne/Becker muscular dystrophy and ASD since his childhood. An elevated level of serum creatine phosphokinase (CPK) was observed, measuring 1068 U/L (normal range/male: 39–308 U/L), along with LDH at 346 U/L (normal range/male:140–280 U/L). His WISC-V report revealed a non-progressive low average score.

Falling within the Composite Score Range of 80–89, he exhibited all three types of specific learning disabilities: dyslexia (reading disabilities), dysgraphia (written communication disorder), and dyscalculia (mathematics problem). While he seemed disheartened by puzzling tasks, his performance on the verbal comprehension index remained within the average range. Socially, he has few close friends, albeit exhibiting immature social skills and displaying repetitive movements and behavior.

#### Subject 4

This proband is a 4-year-old Iranian male with a history of global developmental delay, intellectual disability, muscular dystrophy, and ASD. He was born via Cesarean section after two spontaneous abortions, displaying normal biometric parameters. His parents are consanguineous healthy second cousins. He has hypotonia, specifically notable in his poor head and neck control. He sat independently at 18 months and began walking at 30 months with an abnormal gait. He also has astasia/abasia. From his initial test, his WISC-V score was low falling within the Composite Score Range of 70–79, and this score has not changed. His performance across all WISC-V structures-fluid reasoning, visual-spatial, verbal comprehension, working memory, and processing speed indices-has been consistently reported as low. Although his MRI of the brain and spine was normal, neurobehavioral problems are evident through repetitive behaviors. He has no friends and displays minimal social skills. It is noteworthy that his mother’s thrombophilia test showed abnormalities during pregnancy (the report is unavailable). A muscle biopsy of the proband demonstrated the presence of megaconical congenital muscular dystrophy.

**Table 3:** Clinical characteristics of the four index patients

Case number	Subject 1	Subject 2	Subject 3	Subject 4	
Current age	21	18	16	4	
Sex	F	F	M	M	
Ethnicity	Iran	Iran	Iran	Iran	
Method of detection	ES	ES	ES	ES	
Developmental delay	+	+	+	+	
Intellectual disability	+	+	+	+	
Learning disability	+	+	+	+	
Autism	+	+	+	+	
Epilepsy	-	+	-	-	
Language/speech delay	+	+	-	+	
ADHD	+	+	-	-	
Behavioral problems (repetitive movement and behavior)	+	+	+	+	
Anxiety disorder	+	-	-	NA	
muscular dystrophy	-	-	+	+	
Hypotonia	-	-	+	+	
Impaired motor skills (abnormal gait)	+	-	+	+	
Obesity	-	+	-	-	
Acrophobia	+	-	-	-	

### Molecular analysis with exome sequencing

Our investigation involved ES of 12 families, resulting in the identification of causative genes only in 4 families. Among the remaining 8 families, we identified 6 polymorphic variants in 6 families. Specifically, 3 heterozygous variants were found in one healthy parent within 3 families, while another set of 3 heterozygous variants were found in both healthy parents in an additional 3 families. In the 2 remaining families, we encountered challenges in pinpointing any potential candidate genes. Intriguingly, the investigation led us to the discovery of four variants within known syndromic autism-related genes: *IQSEC2* (c.1637G > A, p.Trp546Ter), *FOXG1* (c.563 C > A, p.Ala188Glu), *DMD* (c.631T > A, p.Leu211Met), and *CHKB* (c.382G > T, p.Glu128Ter).

**Fig. 3:**
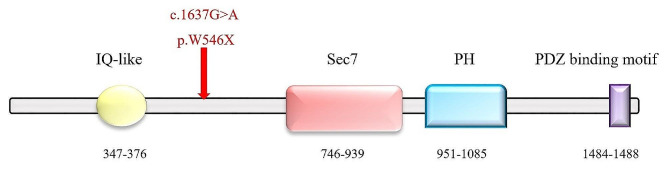
IQSEC2 protein structure and location of the Trp546Ter variant. Schematic representation of the human IQSEC2 protein (NP_001104595), highlighting its IQ-like domain, Sec7 domain, pleckstrin homology domain (PH), and PDZ binding motif. The location of the Trp546Ter variant is indicated in the linker between IQ-like domain and Sec7 domain

For instance, the pathogenic *de novo* variant c.1637G > A in *IQSEC2* (IQ motif and sec7domain2, MIM 300522), as observed in Subject 1, lies between IQ-like and Sec7 domains, situated well before the critical residue for enzymatic activity at position 849. The resulting nonsense variant p.Trp546Ter is predicted to yield a truncated protein devoid of Sec7, PH, and PDZ binding domains if expressed (Fig. 3). Notably, this premature stop codon potentially activate NMD, given its positioning 661 nucleotides (NTs) upstream of the exon 5 and 6 junction (50–55 nucleotide rule) [29]. This scenario could prevent the expression of the truncated protein or, if it does escape NMD, it would lack the prominent Sec 7 enzyme activity, as well as the pleckstrin homology (PH) domain and PDZ binding motif.

**Fig. 4:**
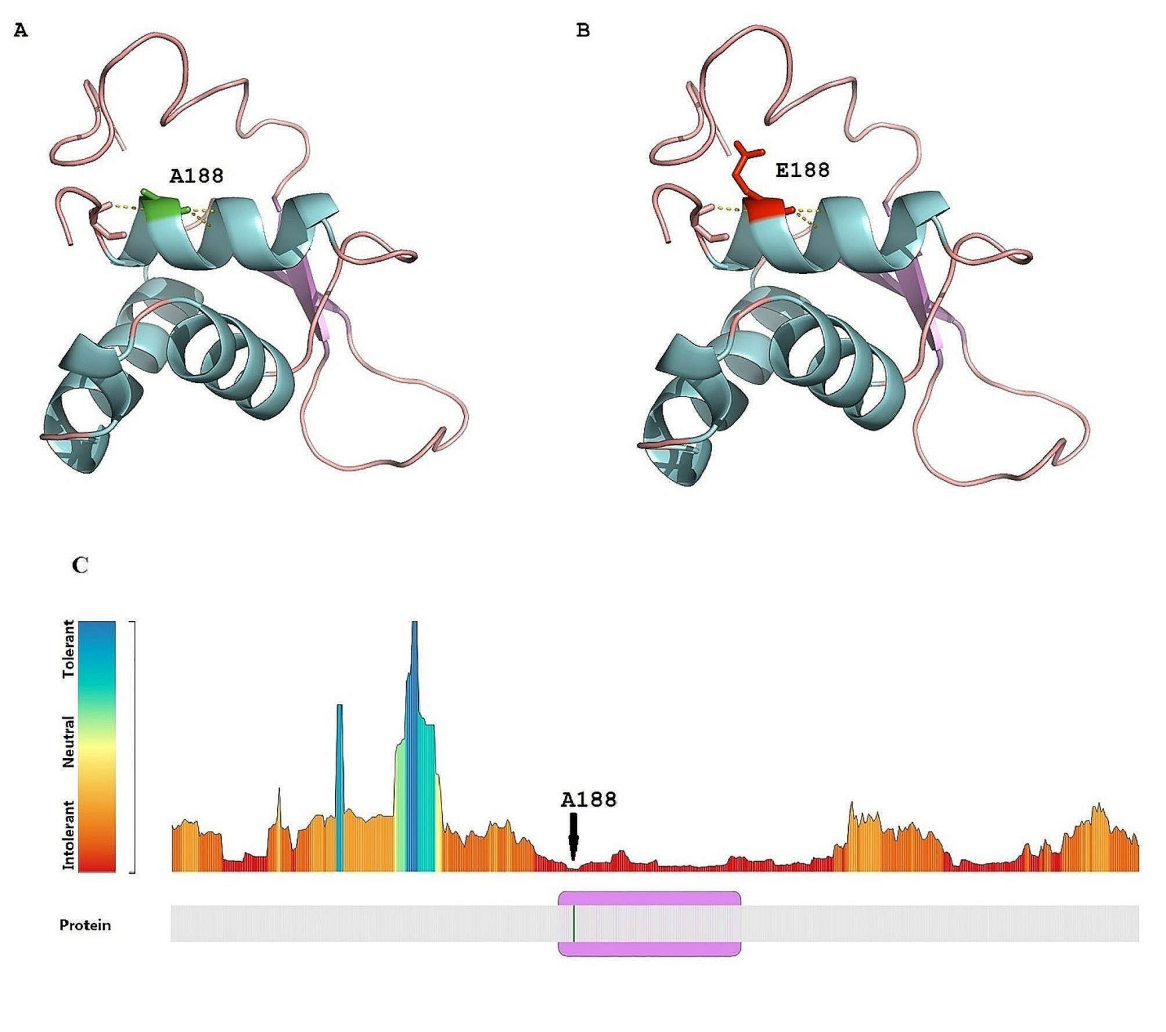
FOXG1 structure and location of *de novo* variant Ala188Glu. A three-dimensional model of FOXG1 amino acids 168–280 (based on PDB 1vtn.1.C) is depicted, showing the overall protein structure. **A** The Alanine residue at position 188 is highlighted in green. **B** The mutated Glutamic acid at position 188 is illustrated in red. The presence of this bulky Glutamic acid likely causes misfolding due to spatial hindrance. **C** Ala188 resides within a highly intolerant region, as identified by MetaDome web server analysis

In Subject 2, an additional notable alteration was a *de novo* heterozygous missense variant, c.563 C > A/ p.Ala188Glu, in *FOXG1* (Forkhead box G1, MIM 164874), affecting a female. This alteration is situated within the forkhead binding domain (FBD, amino acids 179 to 269), known for its high conservation within this single-exon gene (Fig. 4).

**Fig. 5:**
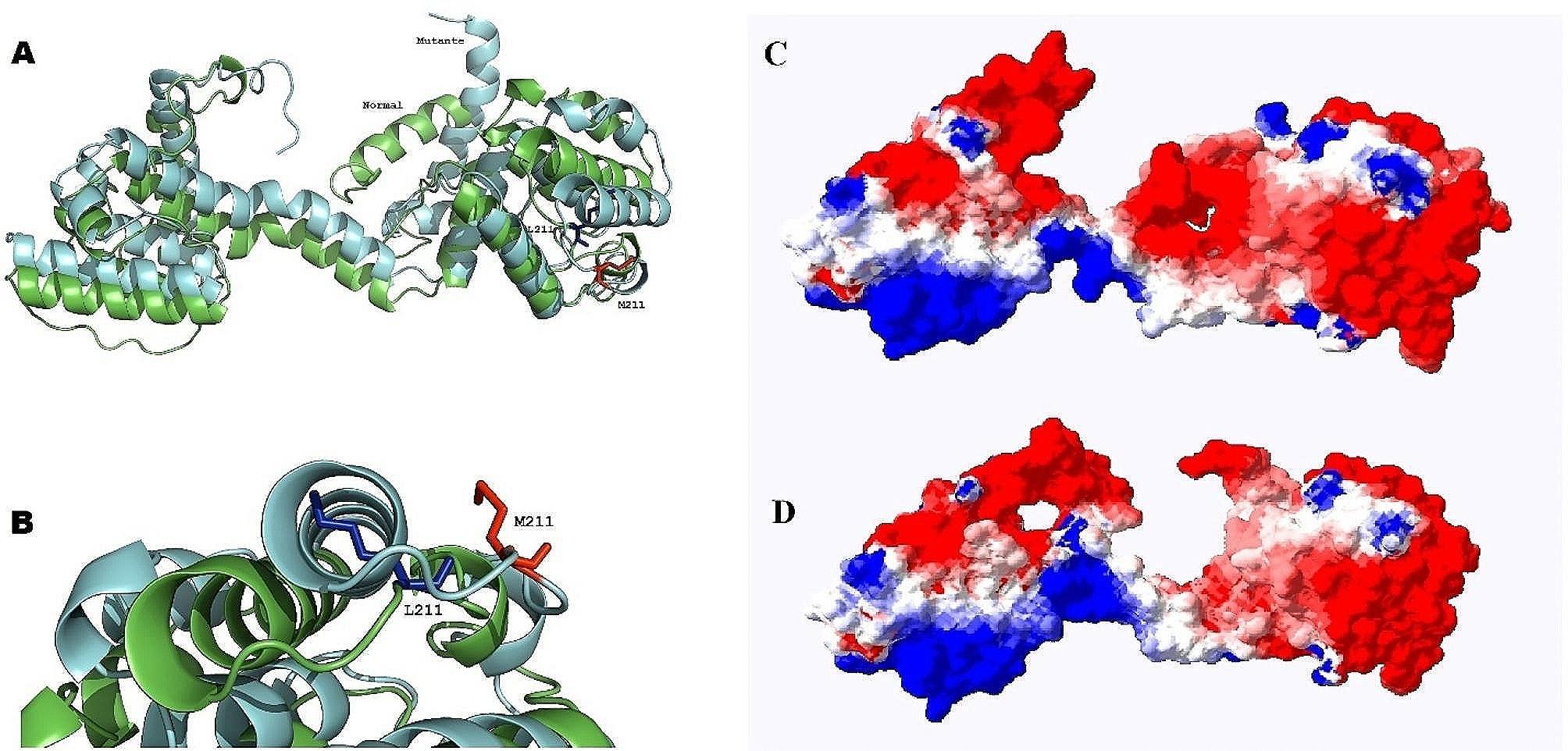
Schematic representation of the Leu211Met substitution in DMD’s N-terminal. **A** Depicts the cartoon structure of both wild-type (in green) and mutated (in cyan) DMD. The presence of a Methionine residue at position 211 induces misfolding within the core of the DMD protein **B** Offers a close-up view of the Leucine (in blue) and Methionine (in red) residues at position 211, highlighting the substitution. **C** Compares the surface electrostaticity of the wild-type DMD and **D** the mutated DMD

Subject 3 displayed a *de novo* hemizygous missense change, c.631T > A, p.Leu211Met in *DMD* (Dystrophin, MIM 300377). This variant was absent in both unaffected parents, and its presence led to a misfolding of the protein’s methionine residue at 211. This alteration in the protein’s 3D structure and surface electrostatic potential has been illustrated through protein modeling (Fig. 5).

**Fig. 6:**
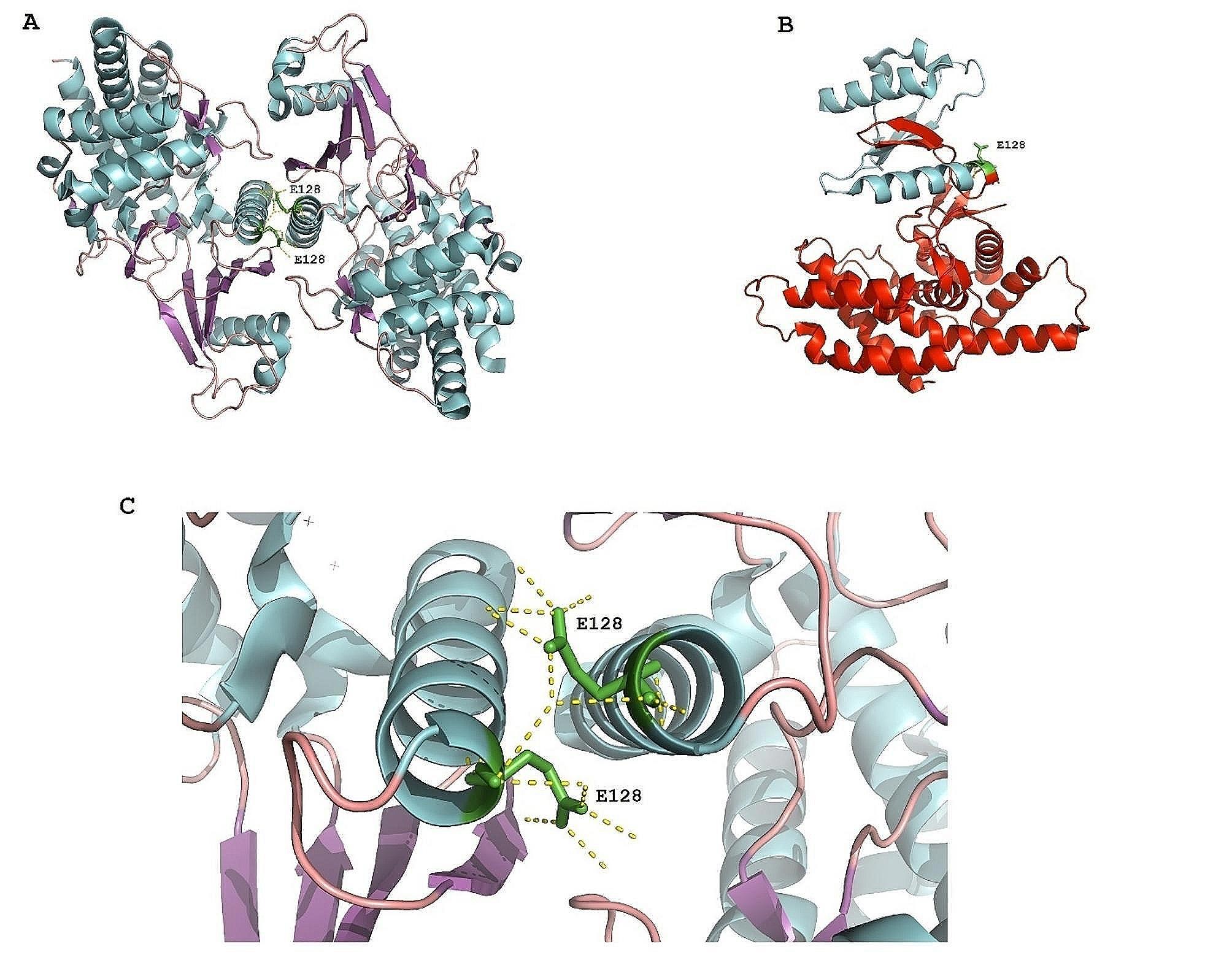
Schematic representation of CHKB structure and glutamic acid at position 128. **A** Illustrates the dimerized CHKB structure (in cyan) with Glutamic acid at position 128 (in green), serving as a negatively charged residue. **B** Emphasizes the significance of the Glu128 residue (in green) for dimerization of CHKB monomers (in red). **C** Provides a magnified view of Figure A, highlighting the crucial role of Glu128 (in green) in CHKB dimerization (in cyan)

Similarly, Subject 4 presented a variant, c.382G > T, p.Glu128Ter, in exon 3 of *CHKB* (Choline Kinase Beta, MIM 612395) with predictions pointing to a truncated protein. This alteration is anticipated to disrupt a substantial portion of the central domain as well as the entire CHKB C-terminal domain. Notably, this variant activates a premature stop codon that may trigger NMD, positioned 66 nucleotides (NTs) upstream of the exon 3–4 junction (50–55 nucleotide rule). [29]. This disruption could prevent the production of CHKB dimers crucial for its choline kinase function (Fig. 6). These findings collectively underscore the significance of the identified variants within well-established autism-related genes and contribute to our broader understanding of the genetic underpinnings of ASD.

### RT-qPCR of four ASD-related genes

**Fig. 7:**
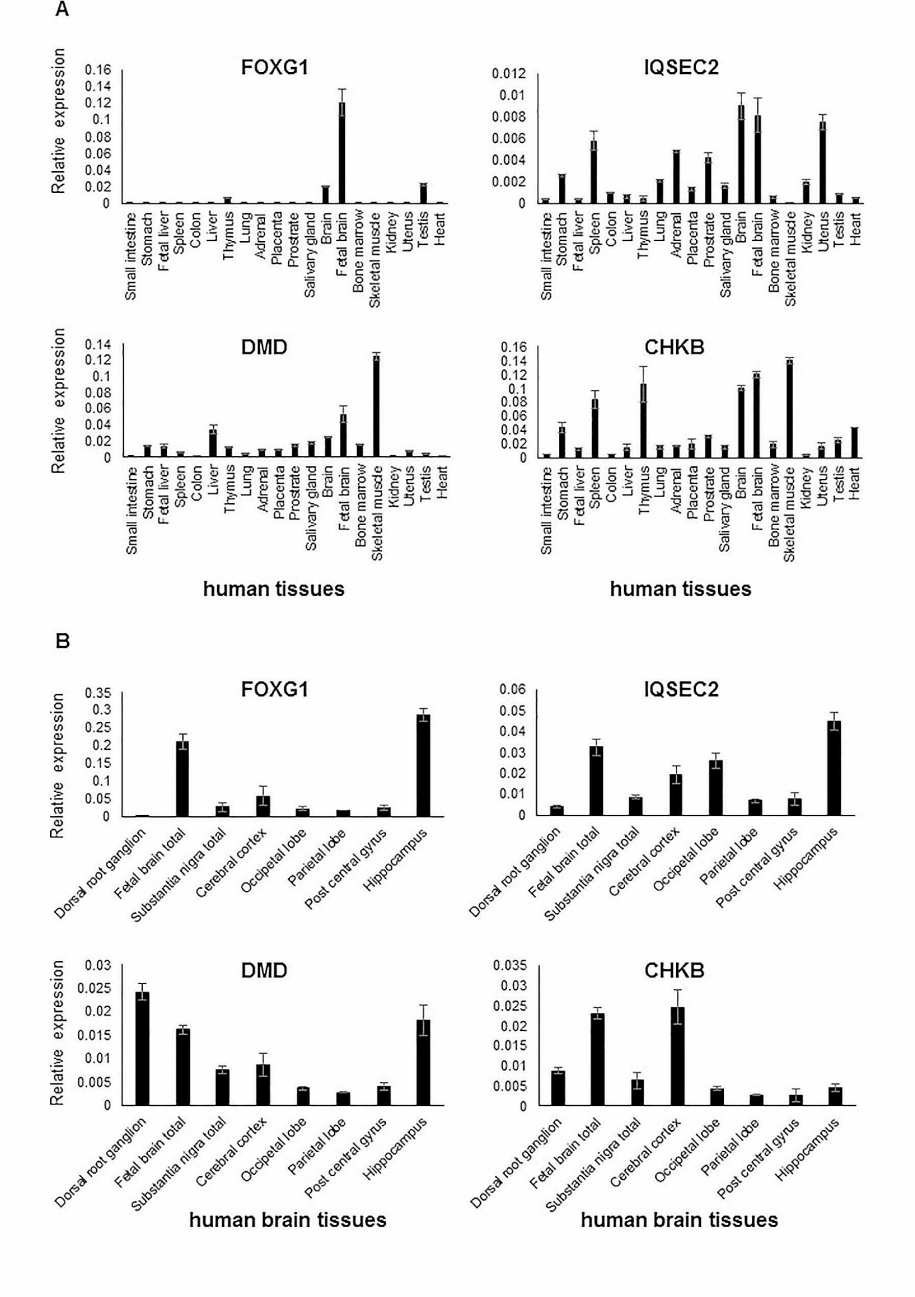
Disease gene transcript levels in human tissues using RT-qPCR. **A** The expression of *FOXG1* and *IQSEC2* is elevated in fetal brain tissue, while *CHKB* and *DMD* expression exhibit their highest expression levels in skeletal muscles. **B** The expression of *FOXG1*,* IQSEC2*,* DMD*, and *CHKB* varies across different parts of the brain, demonstrating distinct levels of expression

We assessed the expression patterns of four ASD-related genes in the brain and various human tissues. Transcript levels of *FOXG1*,* IQSEC2*,* DMD*, and *CHKB* were determined using RT-qPCR. Notably, elevated expression of *FOXG1* and *IQSEC2* was observed in fetal brain tissue compared to other tissues, whereas *CHKB* and *DMD* expressions were most prominent in skeletal muscles (Fig. 7A). All four genes—*FOXG1*,* IQSEC2*,* DMD*, and *CHKB*—exhibited varying expression across different brain regions (see Fig. 7B). High levels of *FOXG1* and *IQSEC2* expression in fetal brain tissues suggest their potential involvement in neurological phenotypes, while increased *CHKB* and *DMD* expression in skeletal muscles points to their roles in muscle disorders.

### Protein modeling

In Subject 2, with an Ala188Glu variant, the Alanine residue at position 188 is located in a region highly intolerant to changes within FOXG1, as indicated by the MetaDome web server (Fig. 4C). The substitution to the bulky Glutamic acid is likely to create spatial and steric hindrance, potentially leading to misfolding of FOXG1 (Fig. 4A and B).

Homology modeling of amino acid 227 in DMD reveals spatial hindrance caused by the mutated amino acid, Methionine, at position 211 in Subject 3, which could result in misfolding of the DMD structure (Fig. 5A and B). Furthermore, the Leu211Met variant leads to alterations in both the three-dimensional structure and the surface electrostatic potential of DMD (Fig. 5D).

The negatively charged glutamic acid at position 128 plays a vital role in the dimerization of the CHKB monomer. Figure 6A and C underscore the significance of Glu128 for CHKB dimerization. Consequently, the nonsense variant p.Glu128Ter at this residue is predicted to be highly deleterious in Subject 4.

### The American College of Medical Genetics and Genomics (ACMG) classification

Following ACMG standards and guidelines [30], we employed standardized terminology to interpret the pathogenicity of genetic variants: ‘pathogenic,’ ‘likely pathogenic,’ ‘uncertain significance,’ ‘likely benign,’ and ‘benign’. In the context of our study involving four variants discovered in Iranian patients, we observed their absence in publicly available population databases such as gnomAD, the 1000 Genome Project, and the Iranome database [18]. This observation provided a moderate level of evidence supporting their pathogenicity (PM2). Utilizing the ACMG criteria for variant classification, we designated all four variants as pathogenic (see Table 4).

### CADD scores

CADD scores for *IQSEC2* (c.1637G > A, NM_001111125.3), *FOXG1* (c.563 C > A, NM_005249.5), and *CHKB* (c.382G > T, NM_005198.4) exceed 30, placing them in the top 0.1% of the most deleterious substitutions [31, 32]. The *DMD* (c.631T > A, NM_004010.3) score was 15, predicting in the top 10% of most deleterious substitutions (see Table 4). These CADD scores align well with the ACMG classification of genetic variants.

**Table 4:** Clinical data, ES results and some prominent related data for defined variants

ID	Subject 1	Subject 2	Subject 3	Subject 4
Gene name	*IQSEC2*	*FOXG1*	*DMD*	*CHKB*
Exon number	exon 5 of 15	exon 1 of 1	exon 10 of 79	exon 3 of 11
Nucleotide change	c.1637G > A	c.563 C > A	c.631T > A	c.382G > T
NM number	(NM_001111125.3)	(NM_005249.5)	(NM_004010.3)	(NM_005198.4)
genomic change (hg38)	g.53,250,939 C > T	g.28,767,842 C > A	g.32,645,113 A > T	g.50,581,814 C > A
NM number	(NM_001111125.3)	(NM_005249.5)	(NM_004010.3)	(NM_005198.4)
Effect on protein	p. Trp546Ter	p.Ala188Glu	p.Leu211Met	p.Glu128Ter
NP number	NP_001104595.1	NP_005240.3	NP_004001.1	NP_005189.2
Alteration type	nonsense	missense	missense	nonsense
ACMG classification	Pathogenic PVS1/PS2/PS4/PM2/PP3	Pathogenic PS2/PS4/PM1/PM2/PM5/PP2/PP3	Pathogenic PS2/PS4/PM2/PP3	Pathogenic PVS1/ PS4/PM2/PM3/PP1/PP3
CADD Score	36	42	15	32
Zygosity	Het	Het	Hemi	Hom
ClinVar	SCV001251727	SCV001337647.1	SCV000746310	SCV000923677
MAF	N/A	N/A	0.0000109	N/A
Inheritance	XLD / *de novo*	AD / *de novo*	XLR / *de novo*	AR
sex	F	F	M	M
Method of detection	ES	ES	ES	ES
Associated Syndrome (MIM)	Intellectual disability, X-linked 1 (MIM 309530) Rett-like syndrome	Rett-like syndrome, congenital variant, FOXG1 syndrome (MIM 613454)	Becker Muscular Dystrophy (MIM 300376) dilated	Megaconial type of congenital muscular dystrophy (CMD) (MIM 602541)

*AF* autism family, *MAF* minor allele frequency, *IP* inheritance pattern, *Hemi* hemizygous, *Het* heterozygous, *Hom* homozygous, *Z* zygosity, *AD* autosomal dominant, *AR* autosomal recessive, *XLR* X-linked recessive, *XLD* X-linked Dominant, *ES* exome sequencing, *NA* not available, *PVS* very strong, *PM* Moderate, *PP* supporting

## Discussion

In consanguineous families with human developmental disorders, autosomal recessive genes are generally identified as genetic etiology. The current study disclosed four disease variants in *IQSEC2*, *FOXG1*, *DMD*, and *CHKB* in four syndromic autism simplex consanguineous families. Accordingly, two *de novo* heterozygous variants were found in *IQSEC2* (c.1637G > A, p.Trp546Ter) and *FOXG1* (c.563 C > A, p.Ala188Glu), respectively. Heterozygous variants in *IQSEC2* and *FOXG1* underlie Rett-like phenotype or atypical Rett syndrome. A novel heterozygous nonsense variant of *IQSEC2* in our female Subject 1 fits well with her diagnosis of atypical Rett syndrome. However, the c.1637G > A variant in *IQSEC2* distinguishes it from the reported variant c.1638G > A, which also leads to the p.Trp546Ter alteration [33]. Importantly, the c.1637G > A variant in Subject 1 is novel at the cDNA level and involves a distinct patient.

In Subject 2, a heterozygous missense variant c.563 C > A, p.Ala188Glu was identified in *FOXG1*, aligning with our clinical diagnosis of Rett-like Syndrome. Notably, this very variant has recently been identified in an Iranian girl of the same age with a similar phenotype [34]. We are currently investigating whether she is the same individual who registered with two different teams.

Interestingly, however, both children are from consanguineous parents. Since *DMD* and *CHKB* are generally known to be involved in muscular dystrophy, a *de novo* novel hemizygous variant in *DMD* (c.631T > A, p.Leu211Met) in Subject 3 and a novel homozygous variant in *CHKB* (c.382G > T, p. Glu128Ter) in Subject 4 imply a potential connection between autism spectrum disorder (ASD) and muscular dystrophy.

Recent studies have highlighted a notable prevalence of ASD in patients with Duchenne Muscular Dystrophy (DMD) and Becker Muscular Dystrophy (BMD). Specifically, these studies have reported ASD prevalence rates ranging from 3.1 to 20.7% in this patient population, which is significantly higher than in the general population [35]. Moreover, Duchenne Muscular Dystrophy has been associated not only with muscle-related symptoms but also with a range of neuropsychiatric disorders, including ASD, attention deficit disorder, hyperactivity, and obsessive-compulsive disorder, underscoring the impact of DMD on brain function as well as muscle function [36].

In the context of *CHKB* mutations, which lead to Megaconial congenital muscular dystrophy, there is a documented association with several neurological and behavioral symptoms. These include intellectual disability and behavioral changes that are related to ASD [37]. Furthermore, individuals with *CHKB*-related muscular dystrophy frequently exhibit conditions such as ASD or ADHD, reinforcing the link between *CHKB* mutations and a spectrum of neurological disorders [38].

These findings collectively highlight the complex link between muscular dystrophy, especially in cases involving *DMD* and *CHKB* gene mutations, and the emergence of autism spectrum disorders. This underscores the importance of addressing both muscular and neurological aspects in the therapeutic approach and comprehensive management of these conditions.

The homozygous nonsense variant, c.382G > T in *CHKB* was heterozygous in the healthy carrier parents. Clearly, this variant follows autosomal recessive inheritance, based on the family history in line with our hypothesis for consanguineous families. Unlike what may be expected, only *CHKB* is an autosomal recessive gene, while *IQSEC2* and *DMD* are X-linked and *FOXG1* is an autosomal dominant gene. Rarely, homozygous variants in autosomal dominant disease genes were previously reported in affected children of consanguineous Iranian families with both parents being healthy, serving as heterozygous carriers [39, 40]. Our findings underscore that consanguineous simplex families could show heterogeneous inheritance patterns, that involve autosomal dominant and X-linked recessive genes in addition to autosomal recessive genes. Non-recessive inheritance in Mid-Eastern simplex consanguineous families was previously reported, but not in Iranian [10].

Among the four novel coding variants, three are *de novo* (two heterozygous variants in *IQSEC2* and *FOXG1*, respectively, and one hemizygous variant in *DMD*). One homozygous variant in *CHKB* was inherited from healthy heterozygous parents. Two missense variants in *FOXG* and *DMD*, respectively, are predicted to affect protein folding, whereas two nonsense variants in *CHKB* and *IQSEC2*, respectively, would likely trigger NMD, preventing their protein expression. When expressed, the nonsense variant in *CHKB* affects its dimerization, which is important for its choline kinase function, and the nonsense variant in *IQSEC2* would eliminate 3 functional domains. As a result, two nonsense (*IQSEC2* and *CHKB*) and two missense variants (*FOXG1* and *DMD*) likely underlie loss-of-function mechanism in these four individuals.

The IQSEC2 protein is localized to excitatory synapses, functioning as a component of the NMDA receptor complex. It interacts with post-synaptic density proteins, including DLG1, DLG2, and DLG4. This positioning and interaction are believed to contribute significantly to synaptic plasticity [41].

*FOXG1* encodes the forkhead box G1 protein, which is a transcription factor. This protein acts as a transcriptional repressor, directly suppressing the transcriptional activity of Zbtb20, Prox1, and Epha4 [42].

Mdx mice contain a nonsense point mutation in *DMD* and thus lacks dystrophin expression in all tissues. The study conducted on this mouse model found altered synaptic transmission and excitability in cerebellar nuclear neurons. The study observed a reduced number of synapses and reduced replenishment rate of synaptic vesicles in Purkinje cells, which could indicate presynaptic dysfunction. These alterations in synaptic function could contribute to cognitive and neurodevelopmental deficits associated with *DMD* [43].

Among the four genes we have identified, *IQSEC2* and *DMD* are implicated in synaptic formation, whereas *FOXG1* is associated with transcriptional regulation. It is noteworthy that both synaptic formation and transcriptional regulation are established pathways significantly involved in the development of autism [4].

*CHKB*, on the other hand, is involved in the synthesis of phosphatidylcholine, a major component of biological membranes. Its role is more aligned with lipid metabolism and cellular membrane synthesis and maintenance, rather than direct involvement of known autism pathways.

In our study, we observed developmental delay, intellectual disability, learning disability, and behavioral disorders as co-existing clinical features in all four patients with autism. Additionally, language/speech delay and impaired motor skills were noted in three of these patients, while two exhibited muscular dystrophy, hypotonia, and ADHD.

In a group of 12 Iranian singlet syndromic autism patients, we identified disease-causing genes in four, achieving a molecular diagnostic rate of 33.3%. Our findings include one autosomal dominant gene (*FOXG1*), one autosomal recessive gene (*CHKB*), one X-linked dominant gene (*IQSEC2*), and one X-linked recessive gene (*DMD*). This contrasts with the inheritance patterns observed in reported multiplex consanguineous autism families, which typically involve autosomal recessive genes.

Notably, both *DMD* and *CHKB* are associated with conditions involving muscular dystrophy and autism. However, the relationship between these two genes in the context of these disorders is not direct but coincidental, as each gene contributes to conditions where muscular dystrophy and autism-like features may manifest through different underlying mechanisms. This highlights the complexity and heterogeneity of both muscular dystrophies and autism spectrum disorders. The distinct disease genes and clinical features observed in our four syndromic autism patients underscore the genotypic and phenotypic heterogeneity present in these Iranian consanguineous singlet families.

## Conclusion

The diagnostic yield in our study is 33.3% (4/12), which is much higher than 13.3% (2/15) of the previous report using 15 simplex consanguineous neurodevelopmental families originated mainly from Syria, Turkey, Egypt, and Jordan [10] or 11.8% of a review of exome sequencing diagnostic yield in simplex consanguineous families [44]. Our study shows that some children with autism may have a genetic defect that affects the muscles, and consanguineous simplex autism individuals might have autosomal dominant or X-linked disease genes. Additionally, future research consisting of animal models and functional studies of these four novel variants are essential to understand the pathological role of these variants.

## Data Availability

The variants identified during the current study are accessible in the ClinVar repository under the following accession numbers: SCV001337647.1 (*FOXG1*), SCV001251727.1 (*IQSEC2*), SCV000923677.1 (*CHKB*), and SCV000746310.2 (*DMD*).

## Abbreviations

ACMG: American College of Medical Genetics
ADHD: Attention deficit hyperactivity disorder
ARFGEF: ADP-Ribosylation Factor (ARF) family of small GTP-binding proteins
ASD: Autism Spectrum Disorder
BWA: Burrows-Wheeler Aligner
CCGS: Center for Comprehensive Genetic Services
CMD: Congenital muscular dystrophy
CMA: Chromosomal microarray
CNS: Central Nervous System
CNV: Copy number variant
DGC: Dystrophin-glycoprotein complex
DPC: Dystrophin associated protein complex
DSM-V: Diagnostic and Statistical Manual of Mental Disorders − 5th Edition
ES: Exome Sequencing
gDNA: Genomic DNA
MAF: Minor Allele Frequency
NDD: Neurodevelopmental disorders
NGS: Next Generation Sequencing
NMD: Nonsense mediated decay
PCR: Polymerase Chain Reaction
PSD: Postsynaptic density
SBMU: Shahid Beheshti University of Medical Sciences
VUS: Variant of Uncertain Significance
